# Visual Impairment Spatial Awareness System for Indoor Navigation and Daily Activities

**DOI:** 10.3390/jimaging11010009

**Published:** 2025-01-04

**Authors:** Xinrui Yu, Jafar Saniie

**Affiliations:** Department of Electrical and Computer Engineering, Illinois Institute of Technology, Chicago, IL 60616, USA; xyu47@hawk.iit.edu

**Keywords:** indoor positioning, indoor navigation, object recognition, visually impaired

## Abstract

The integration of artificial intelligence into daily life significantly enhances the autonomy and quality of life of visually impaired individuals. This paper introduces the Visual Impairment Spatial Awareness (VISA) system, designed to holistically assist visually impaired users in indoor activities through a structured, multi-level approach. At the foundational level, the system employs augmented reality (AR) markers for indoor positioning, neural networks for advanced object detection and tracking, and depth information for precise object localization. At the intermediate level, it integrates data from these technologies to aid in complex navigational tasks such as obstacle avoidance and pathfinding. The advanced level synthesizes these capabilities to enhance spatial awareness, enabling users to navigate complex environments and locate specific items. The VISA system exhibits an efficient human–machine interface (HMI), incorporating text-to-speech and speech-to-text technologies for natural and intuitive communication. Evaluations in simulated real-world environments demonstrate that the system allows users to interact naturally and with minimal effort. Our experimental results confirm that the VISA system efficiently assists visually impaired users in indoor navigation, object detection and localization, and label and text recognition, thereby significantly enhancing their spatial awareness and independence.

## 1. Introduction

According to the Global Vision Database 2019 Blindness and Vision Impairment Collaborators, the year 2020 saw approximately 43.3 million people living with blindness, and another 295 million people experiencing moderate to severe vision impairments. Projections suggest a significant increase by 2050, with the blind population expected to rise to 61.0 million, and those with moderate to severe vision impairments expanding to 474 million individuals [1]. In the United States alone, there were more than one million blind people in the year 2015, and that number is projected to double in the year 2050 [2]. These statistics highlight an escalating global health concern that necessitates immediate attention and action. There can be no overstatement about the importance of vision. It is a fundamental sensory modality that underpins a myriad of daily activities, including but not limited to navigation, fetching objects, reading, and engaging in other complex tasks, all of which are integral to personal independence and quality of life [3,4,5].

While there is no single most important task above all others, navigating indoor spaces poses a unique set of challenges for visually impaired individuals, often complicating what many would consider routine activities [6]. Addressing this task, both individually and collectively, is central to empowering visually impaired individuals to complete not only basic but also complex tasks with greater confidence and autonomy. Even before the advent of computer vision, many methods, tools, and systems were developed to assist the visually impaired in navigation. Some common examples include white canes, guide dogs, and braille. However, without machine vision and AI technologies, these methods were inherently limited. A white cane, for instance, while invaluable for immediate spatial detection, offers a limited range and no identification capabilities [6]. Guide dogs, often considered the best alternative to sighted assistance, offer companionship, increased mobility, and sometimes even higher social status [7,8]. Yet, they come with high training and acquisition costs, making them mostly unavailable for low-income individuals [9]. Also, despite their ability to navigate complex environments, guide dogs cannot communicate specific facility information to their handlers [10]. Braille has revolutionized access to written information for the visually impaired, but it is confined to touching and needs to be printed beforehand, limiting its capacity to deliver immediate and dynamic content [11].

**Figure 1:**
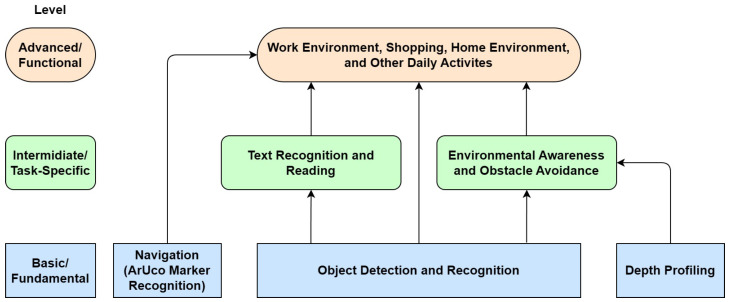
Layered approach to holistic assistance for visually impaired individuals.

With the rapid advancements in the field of computer and machine vision, the landscape of technologies to assist the visually impaired is undergoing a transformation. The advent of text-to-speech (TTS) and speech-to-text (STT) technologies can greatly improve the interfacing options for the visually impaired [12]. Coupled with the emergence of deep learning algorithms, tasks such as object recognition, which were once challenging, are now attainable and can be integrated into practical applications. Moreover, the progress in embedded systems and system-on-chip (SoC) technologies heralds the advent of portable and wearable smart devices tailored to the needs of the visually impaired [13]. However, many existing systems, including AI-based ones, only specialize in a single aspect of assistance, with the majority of the systems reviewed aiming to solve only one of three tasks: object recognition, obstacle avoidance, and navigation [14,15]. As a result, a holistic system that can seamlessly integrate various functionalities—from navigation assistance to object and text recognition—can be greatly beneficial for visually impaired users. Our proposed VISA system utilizes cutting-edge AI technologies, including advanced object detection and spatial navigation algorithms, alongside user-friendly interfaces to assist visually impaired users in overcoming the challenges associated with indoor activities. By focusing on this area, the proposed VISA system aims to provide a comprehensive solution that can be adapted and expanded to meet a wide range of needs and activities, ultimately facilitating a more accessible and navigable environment for visually impaired individuals. The integration of the tasks is shown in Figure 1.

**Figure 2:**
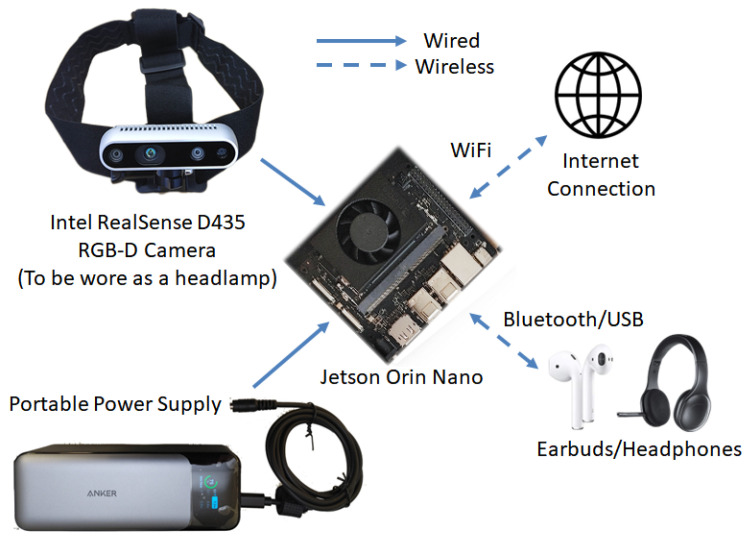
VISA system diagram.

A system diagram of our Visual Impairment Spatial Awareness (VISA) system is shown in Figure 2. We selected the NVIDIA Jetson Orin Nano as the core of the VISA system due to its optimal balance of power efficiency, compact size, and good computing performance. We chose an Intel RealSense D435 RGB-D camera since its specifications suit indoor navigational use and it has a compact size. While the Internet connection is shown on the diagram, all essential functionalities are completed locally. More detailed discussions regarding hardware selection and configuration are given in Section 3, Section 4 and Section 5.

## 2. Review of Existing Indoor Navigation Technologies

Indoor navigation presents a unique set of challenges that are distinct from outdoor navigation. GPS, which is commonly used for outdoor navigation, is often ineffective indoors due to the lack of satellite signals. As a result, various indoor navigation systems have been developed to assist visually impaired individuals in navigating indoor environments [16,17,18,19]. Those systems can be classified into two groups according to the reliance on external components or pre-installed infrastructures: networked and self-contained. The networked group consists of radio frequency identification (RFID), near-field communication (NFC), ultra-wideband (UWB), Bluetooth low energy (BLE), and infrared. The self-contained group consists of single, stereo (dual), and RGB-D cameras, together with lidar. It should be noted that fiducial markers like QR (quick response) codes [20] and AR (augmented reality) markers, namely ArUco (Augmented Reality University of Cordoba) markers [21], can be used in conjunction with cameras. The markers are passive, i.e., they do not communicate with the system, and are thus still included in the self-contained group.

### 2.1. Networked Navigation Systems

RFID technology employs RFID tags and a reader device such as a smartphone. Users of such a system need to carry an RFID reader as they navigate, while RFID tags containing identification information are placed at locations of interest. Upon reaching a specific location and reading the corresponding RFID tag, the read data facilitate further information retrieval from a database and assist in accurately positioning the user within a map [16]. While there are active RFID tags that supports maximum ranges of 40 m to 1 km [22], passive RFID tags with a maximum range of 0.5 m to 10 m may see more usage [23], as they do not require setting up a power supply infrastructure for the active RFID tags.

NFC shares some common properties with RFID and can be considered part of the RFID family in some cases. Most smartphones nowadays have built-in NFC reader modules, saving the need a separate NFC reader device to be carried by the visually impaired user. While there exist NFC based applications for the visually impaired [24], the technology is severely hampered by the short range of NFC communication. In the best-case scenario, NFC is limited to operations within several centimeters, which makes any practical applications challenging [16].

UWB technology uses radio waves with a very low energy level for short-range, high-bandwidth communications over a relatively large portion of the radio spectrum. An important advantage of UWB over RFID is that it does not need line-of-sight (LoS) communication, making it usable in complex indoor environments. However, active beacons are needed for UWB. UWB is also accurate enough even for indoor applications, with an accuracy of 0.15 m at a 95% confidence interval. With an operating range of 90 m in low-data-transfer mode, its range is sufficient for indoor navigation in large buildings. UWB is applied in [25] to create an indoor navigation system called SUGAR (Sistema Universal de Guiado Avanzado en Recintos cerrados, Universal Advanced Guidance System in Enclosed Areas) for visually impaired users. The authors claimed that this system has high accuracy and low installation complexity.

BLE is a power-conserving variant of Bluetooth, designed for short-range communication with low energy consumption. Similar to UWB, it can be used to create a network of beacons for indoor navigation. Compared with passive RFID and NFC, it offers a larger operational range of up to 75 m, thus requiring fewer beacons to cover the same area. An indoor navigation system called GuideBeacon is presented in [26], which permits visually impaired users to interact with pre-deployed Bluetooth-based beacons with their smartphones for indoor navigation. The system is able to locate the users accurately in areas of interest.

Finally, infrared-based navigation systems also use the same reader–receiver setup as is in BLE and UWB. However, it does need line-of-sight communication. One example is [27], which uses 16 infrared transmitters in an indoor environment to send infrared signals. The user wears a cap with an infrared receiver and processing unit, obtaining positional information by analyzing received signals. It should be noted that there exists a reversed setup, where the infrared beacons or emitters are placed on the user instead of the surrounding environment, and receivers placed at different locations will receive the emitted infrared signals to locate the user [28].

To summarize, networked systems harness external connections and infrastructure to deliver extensive navigation solutions. Incorporating technologies such as RFID, NFC, UWB, BLE, and infrared, they supply visually impaired users with positional information for navigation. However, the dependency on external components presents notable disadvantages, including higher cost, potential connectivity issues, and problems involved in setting up the infrastructure. These challenges may limit the applicability and effectiveness of networked systems in certain environments, especially for systems that require active distributed components. A comparison of different networked systems is included in Table 1.

#### Self-Contained Navigation Systems

Networked navigation systems often rely on pre-installed infrastructure such as RFID tags or Bluetooth beacons. However, the installation of additional infrastructure may not be feasible in all environments. In the development of assistive technologies for visually impaired individuals, self-contained systems present a distinct approach to navigation, diverging from the dependency on the external infrastructure characteristic of networked systems. Self-contained systems are defined by their autonomy, carrying all necessary hardware to perceive and navigate through spaces independently. They rely on onboard sensors and processing to understand and interact with the environment. This section explores different technologies used in self-contained systems, including single, stereo (dual), and RGB-D cameras, as well as LiDAR, and their integration with passive fiducial markers like AR markers and QR codes. A comparison of these technologies in terms of operation principle, range, accuracy, cost, and facts worth noting is provided in Table 2. Again, it should be noted that the systems that use fiducial markers are still included in this category, primarily because the fiducial markers differ from other necessary infrastructure components due to their passive nature, exceptional cost-effectiveness, and minimal to total lack of maintenance requirements.

Single-camera systems offer a straightforward and cost-effective means to capture visual data, though they are limited by a lack of depth perception. While it is possible to perform monocular depth estimation with a single camera [29], the accuracy and reliability of such depth estimation are mostly insufficient for indoor navigation. As a result, the majority of indoor navigation systems with a single camera need to work in conjunction with fiducial markers, like AR markers and QR codes. As shown in [30], the QR codes can still be easily detected under low-light conditions and a 60% blurriness ratio, making these systems suitable for application indoors and while moving. Examples using ArUco markers exist as well [31,32]. ArUco markers, in comparison to QR codes, store less information and offer fewer unique variants in their most commonly used formats. In return, with the same physical size, they excel in being recognizable from greater distances and at wider angles of incidence [32]. Another system uses a different type of AR marker to register the indoor environment and acquire orientation information [33]. In terms of recognition distance and angle of incidence, this type of AR marker occupies an intermediate position, offering a balance between QR codes and ArUco markers. However, as the angle of incidence becomes more extreme, the minimum size required for AR markers to remain recognizable increases exponentially, which may hinder their deployment. It should be noted that there exist single-camera systems that do not use fiducial markers, like in [34].

Stereo camera systems use dual cameras for depth perception. Mimicking human binocular vision, these systems calculate the depth from the disparity between images captured by two spatially separated cameras, significantly enhancing depth accuracy. One early example is given in [35]. It uses an optical flow-based algorithm [36,37], which aims to calculate the motion between two image frames, and does not rely on fiducial markers for navigation. Still, it should be noted that although a stereo camera system can be used for accurate depth measurement, the related hardware is sometimes expensive and not as widespread as single-camera hardware [38]. With the improvements in modern electronics, it is possible to use embedded systems as the core of such stereo camera systems [39], but such systems are more widely used for obstacle detection [40,41,42] rather than navigation.

RGB-D cameras are imaging devices that capture both color information (RGB) and depth data (D) for each pixel, providing a comprehensive three-dimensional view of the environment. This dual capability provides a comprehensive understanding of spatial relationships, necessary for various applications, including navigation assistance for visually impaired individuals. Over the past decade, there has been a significant amount of research focused on developing navigation systems that leverage RGB-D cameras. At first, there were no RGB-D cameras dedicated to the task, so many studies were carried out using the Microsoft Kinect Camera [43,44,45,46,47]. It is worth mentioning that AR marker-based navigation can be used in conjunction with an RGB-D camera as well [47]. While the Kinect Camera has good depth profiling capabilities within a few meters, it is about a foot long and not convenient to be worn by a user. As more models of RGB-D cameras have been made available on the market, we have seen navigation assistants developed with different cameras. In [48,49], researchers used Google Tango devices, which have built-in RGB-D cameras. Researchers used an ASUS Xtion Pro in [50], and an Intel RealSense D435 was used for [51]. In general, more RGB-D cameras that are suitable for the task of indoor navigation have been introduced in terms of size, weight, and power requirements. Also, some systems have integrated depth information with deep learning algorithms, enhancing their abilities to recognize different types of obstacles, thus leading to more efficient navigation [52].

Lidar (Light Detection and Ranging) devices utilize laser pulses to measure distances to surrounding objects, creating precise 3D maps of the environment. This technology is particularly effective for identifying small obstacles and accurate recognition. Its ability to function effectively in a variety of lighting conditions, from bright daylight to complete darkness, further underscores lidar’s versatility as a navigation aid. Numerous lidar-based systems for autonomous driving have been developed and put to the test [53,54]. A recent review paper [55] highlighted numerous studies that have explored the use of lidar technology for indoor and outdoor navigation to assist the visually impaired. In addition to conventional rotating lidar, there are systems that utilize a lidar sensor integrated into a smartphone, eliminating the need for dedicated devices and reducing the amount of additional hardware the user must carry [56]. Another system was developed with two single-point lidar sensors, saving costs on typically expensive lidar components that contain motors for 360-degree coverage [57]. It should be noted that rotating lidar is better suited for wheeled platforms, and not suitable to be worn on a human user.

To summarize, self-contained systems stand out for their independence from external infrastructure that uses active components. This autonomy ensures that visually impaired users can depend on these technologies for guidance, regardless of the availability of networked components. Moreover, the use of advanced imaging and sensing technologies allows for a richer interpretation of the environment, facilitating more informed and safe navigation decisions. Expanding on this, the adaptability of self-contained systems to various environments without the need for connectivity or external data inputs highlights their versatility and robustness. This capability is particularly important in areas where network infrastructure is limited or non-existent, or cannot be installed, ensuring that the benefits of assistive technologies are accessible to a broader range of visually impaired individuals. Ultimately, the development and refinement of self-contained systems aim to empower users with greater autonomy and confidence in navigating their surroundings, significantly enhancing their quality of life. A comparison of different self-contained systems is included in Table 2.

The exploration of self-contained versus networked systems highlights an important consideration in the development of assistive technologies for visually impaired individuals. While networked systems benefit from the scalability and specificity provided by external infrastructure, self-contained systems offer unparalleled reliability and versatility, essential attributes for enhancing the independence and mobility of visually impaired users. As research in this field progresses, the integration of these systems and their technologies promises to drastically improve accessibility and spatial awareness for the visually impaired community.

**Table 2:** Comparison of self-contained navigation systems for the visually impaired.

Feature/Sensor Type	Single Camera	Stereo Camera	RGB-D Camera	Lidar
**Principle of Operation**	Marker Recognition [29]	Marker Recognition + Depth Perception [35]	Marker Recognition + Depth Perception	ToF Sensor + SLAM [53]
**Typical Range**	Depends on marker size and type [30]	Depends on marker size and type [30]	Depends on marker size and type [47]	Up to 90 m [53]
**Accuracy**	Low to Moderate [31,32]	Moderate	Moderate [45,46] to High [50,51]	High [53]
**Cost**	Low	Low to Moderate	Moderate	High [57]
**Notes**	Needs fiducial markers (also used for range estimation) [31,32]	Needs fiducial markers	Needs fiducial markers	No infrastructure needed; rotating ones not suitable for carrying [56]

## 3. Object Recognition and Localization

**Figure 3:**
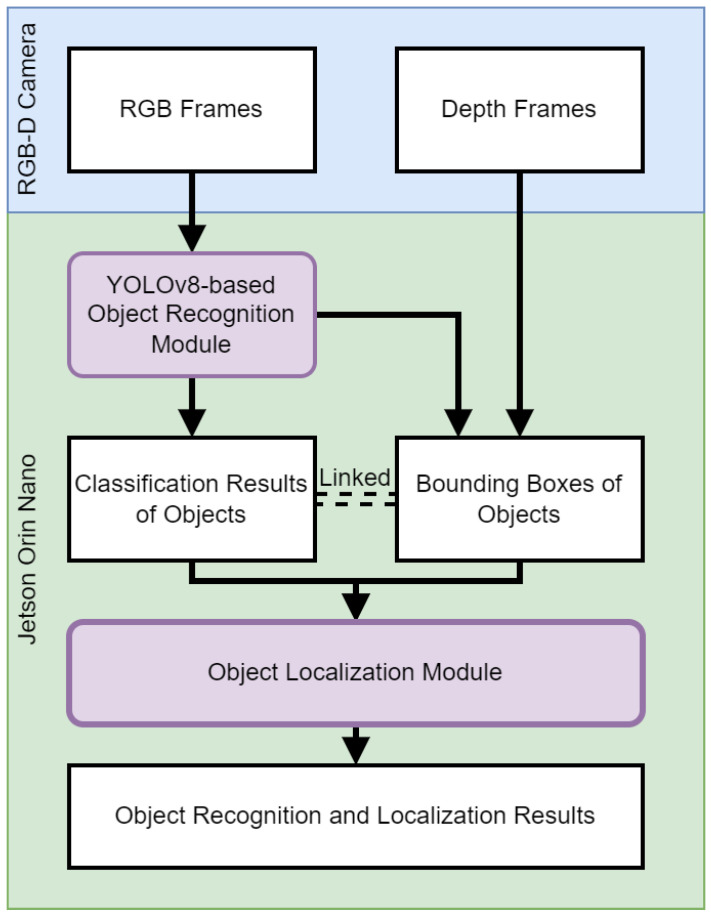
Flowchart of object recognition and localization.

This section discusses the object recognition and localization module in our VISA system, pivotal in assisting visually impaired users by enabling them to identify and pinpoint the location of everyday objects in their vicinity. At the heart of this exploration is the deployment of a sophisticated RGB-D camera system, paired with the cutting-edge capabilities of YOLOv8—a state-of-the-art neural network model renowned for its accuracy and speed in object recognition tasks. This section aims to dissect the technical underpinnings of the object recognition and localization module, providing a comprehensive overview of the module’s architecture, and the integration of depth sensing to augment spatial awareness. A flowchart of the object recognition and localization processes is shown in Figure 3.

### 3.1. Vision-Based Real-Time Object Recognition

**Figure 4:**
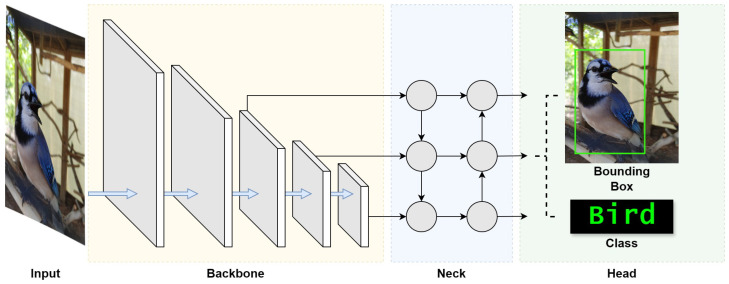
YOLOv8 model structure [60].

The object recognition module is the linchpin of our VISA system, endowed with the advanced capabilities of the YOLOv8 algorithm. This incarnation of the YOLO series [58] is a state-of-the-art object detection model that has been trained on the COCO (common objects in context) dataset, which encompasses an array of 80 object classes ranging from everyday household items to complex environmental elements [59]. The model structure of YOLOv8 is shown in Figure 4. This is drawn on the basis of [60].

To optimize real-time object recognition for visually impaired users, ensuring swift and precise assistance in diverse environments, it is important to understand YOLOv8’s architecture. To begin, we look into the three fundamental blocks of the architecture, namely the backbone, neck, and head, which together facilitate the entire object recognition process.

The backbone block works as the feature extractor of the YOLOv8 model, and is the first to perform operations on the input image. This block is tasked with identifying and extracting meaningful features from the input image. Starting with the detection of simple patterns in its first few layers, the backbone progressively captures features at various levels, enabling the model to construct a layered representation of the input with sufficient extracted features. Such detailed feature extraction is crucial for the understanding required in object detection.

The neck block follows the backbone block, which acts as an intermediary between the feature-rich output of the backbone block and the head block that generates the final outputs. The neck block enhances the detection capabilities of the model by combining features and taking contextual information into account. It takes feature extractions from different layers of the backbone block, effectively creating layered feature storage. This process allows the model to detect objects large and small. This section of the network also works to streamline the extracted features for efficient processing, striking a balance between speed and the accuracy of the model’s output.

The final block, the head, is where the results of the object detection process are generated. Utilizing the layered features prepared by the neck block, the head block is responsible for categorizing, producing bounding boxes, and assigning confidence levels for each detected object. This part of the network encapsulates the model’s ability to not only locate but also identify objects within an image, making it a vital block in the YOLO architecture. Through the coordinated functioning of these three blocks, YOLO achieves its objective of fast and accurate object detection.

In addition, the convolutional nature of YOLOv8 should be examined. The YOLO architecture performs feature analysis on a local level, focusing on specific regions of an image rather than analyzing it in its entirety. The method relies heavily on the repeated application of convolutions throughout the algorithm to generate feature maps, useful in enabling efficient real-time operation.

YOLOv8 provides a total of five models with different numbers of model parameters: YOLOv8n (Nano), YOLOv8s (Small), YOLOv8m (Medium), YOLOv8l (Large), and YOLOv8x (eXtreme). Their corresponding model parameters are shown in Table 3. Based on the trade-off between accuracy and efficiency, we chose YOLOv8s as the model to be used in our VISA system. It has the second-fewest parameters at 11.2 million [58]. YOLOv8s represents an option in the YOLO lineage that is suitable for embedded systems and edge computing, providing a model that is optimized for operational efficiency but still sufficiently accurate. This balance is crucial for real-time applications such as our VISA system, which runs on the NVIDIA Jetson Orin Nano—a platform known for its balance of power and performance in edge computing scenarios. According to the research carried out in [58] and our test result in Table 3, we conclude that YOLOv8s’ position is at the sweet spot of the trade-off between inference time and performance. In other words, a simpler network model like YOLOv8n leads to an unacceptable accuracy drop with no applicable increase in FPS (frames per second), while a more complex model like YOLOv8m reduces FPS noticeably with little improvement in accuracy. Such equilibrium ensures that our VISA system can deliver the real-time object recognition necessary for the navigation and interaction of visually impaired users, while not sacrificing recognition accuracy.

The COCO dataset, the training ground for YOLOv8s, is instrumental in the model’s ability to discern a diverse set of objects. This large-scale dataset facilitates the model’s learning and generalization capabilities, making it robust against the varied visual scenes encountered in indoor environments. The training process involves exposing the model to numerous annotated images, allowing it to learn the features and characteristics of different objects, which leads to the reliable performance of our VISA system.

Implementing YOLOv8s within our assistive technology involved leveraging the pre-trained model and adapting it to the system’s requirements. By integrating the model with the RealSense camera, we crafted a real-time feedback loop that processes visual data to inform and guide users. The module, thus, interprets the class information of recognized objects, to be used for the object localization module and other modules in the VISA system.

**Figure 5:**
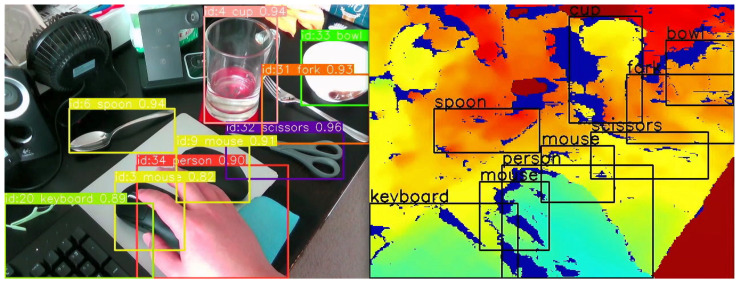
Object recognition results (**left**) and overlay on the depth image (**right**).

Our empirical tests have demonstrated that YOLOv8s maintains its robust performance in real-world scenarios pertinent to our VISA system. The tests involved running the model through a series of indoor environments, capturing its detection capabilities, and measuring the latency and accuracy of its responses. These tests confirmed the model’s aptness for the intended use-case, ensuring that visually impaired users receive timely and precise information about their surroundings. A comparison of different YOLOv8 models running on the Jetson Orin Nano platform is given in Table 3. The test confirms our statement of the sweet spot for YOLOv8s, as it achieved great average FPS and low power consumption, while not suffering from low accuracy. The object recognition results are shown in Figure 5, which indicates accurate recognition of everyday objects indoors.

The YOLOv8s object recognition module is a testament to the advancements in machine learning and its applications in assistive technologies. By leveraging the cutting-edge capabilities of YOLOv8s, our VISA system represents a significant step forward in providing visually impaired individuals with greater autonomy and a more profound interaction with their environment. The module’s ability to process complex visual data in real time opens new avenues for research and development in assistive technology, promising a future where such systems are not just aids but integral parts of how individuals with visual impairments engage with the world around them. Also, the information obtained by the YOLOv8s object recognition system will be utilized by the object localization module, namely recognized object classes and bounding boxes. An example is shown in Figure 5, where we overlay the object classes and bounding boxes onto the depth image. We shall discuss the localization module in the next section.

### 3.2. Object Localization and 3D Visualization

**Figure 6:**
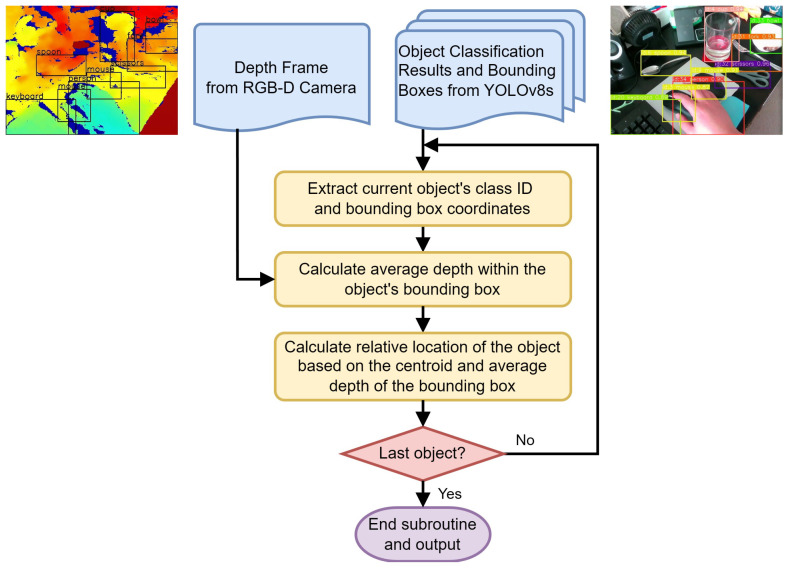
Flowchart of the object localization module.

The object localization and 3D visualization module is a pivotal component of our VISA system, designed to translate the object classification results and depth data into locations of the objects in a three-dimensional space. This module uses the bounding boxes and class names provided by the object detection module to generate spatial awareness, enabling visually impaired users to engage with their environment more effectively. A flowchart of the object localization module is shown in Figure 6.

The object localization module, as depicted in Figure 6, performs a series of steps to identify the location of objects for visually impaired individuals within a 3D environment, on a frame-by-frame basis. The inputs to this module include a depth frame from the RGB-D camera and the corresponding object classification results, which include bounding boxes around detected objects. The classification results act as a backdrop for correlating additional information for other modules, such as the navigation module and the text-to-speech module.

To begin processing, the module extracts the class ID and the bounding box coordinates for each detected object. The class ID indicates the type of object, while the bounding box coordinates define its location in the camera’s field of view.

A key step in this module is the accurate calculation of the average depth within the bounding boxes. The RealSense D435 RGB-D camera utilizes the left image sensor as the reference for the stereo-matching algorithm to generate depth data, resulting in a non-overlapped region in the camera’s depth frames. This non-overlapped region (at the left edge of the frame and objects) contains no depth data (all zeros). Examples of the non-overlapped regions can be seen in the right part of Figure 5, shown as regions in deep blue. The module masks out all such values within the depth frame that fall inside the object’s bounding box. By doing this, we ensure that the average depth represents the true distance to the object.

The average depth within the masked bounding box is then calculated, which provides an estimation of how far the object is from the camera. This step is essential in determining the distance to the object, which is necessary information for a number of different modules, including but not limited to navigation, obstacle avoidance, and the human–machine interface. It can be used to inform the user of the proximity of objects, enhancing their spatial awareness and aiding in safe navigation.

After calculating the average depth, the module computes the object’s relative location based on the centroid of the bounding box and the previously determined average depth. This step ascertains the object’s position in three-dimensional space relative to the camera, providing spatial orientation in the form of the azimuth, the elevation, and the depth of the object. A detailed description of the calculation for azimuth and elevation is given in Section 3.2.1. Finally, a decision step checks if the current object is the last one in the list for this frame. If not, the process loops back to handle the next object. If it is the last object, the subroutine ends.

Upon completion, the module outputs the processed data, which include the distance and relative location of all detected objects within the camera’s field of view. This output can then be used to inform visually impaired users about their immediate surroundings or to guide navigation systems in real time. It also serves as a foundation for translating the results into other sensory modalities, such as audio feedback.

This entire process is optimized for real-time operation, acknowledging the necessity for immediate feedback in an assistive context. The module is fine-tuned to work in concert with the object detection module, ensuring that the visualizations it produces are both current and relevant to the user’s immediate surroundings.

#### 3.2.1. Azimuth and Elevation Calculations

For precise object localization and to obtain data for preventing collisions, it is essential to compute the azimuth and elevation of objects identified within the field of view of an RGB-D camera. This calculation necessitates knowledge of the camera’s field of view and its resolution, specifications that depend on the camera model and can typically be found in its data sheet. The equation to ascertain an object’s azimuth, or its relative horizontal positioning, employs the following parameters: θ=xα/W−α/2. Here, *x* is the horizontal position of the geometric center of the region, in terms of the number of pixels from the left edge of the image; α is the horizontal field of view of the depth camera in degrees; *W* is the resolution of the image along the horizontal axis; and θ is the azimuth of the obstacle in degrees, with 0 indicating dead ahead, a negative value indicating to the left, and a positive value indicating to the right.

Similarly, the following equation can be used to determine the relative vertical position (elevation) of an object: ϕ=yβ/H−β/2. Here, *y* is the vertical position of the geometric center of the region, in terms of the number of pixels from the top edge of the image; β is the vertical field of view of the depth camera in degrees; *H* is the resolution of the image along the vertical axis; and ϕ is the elevation of the obstacle in degrees, with 0 indicating dead ahead, a negative value indicating below the horizon, and a positive value indicating above the horizon.

**Figure 7:**
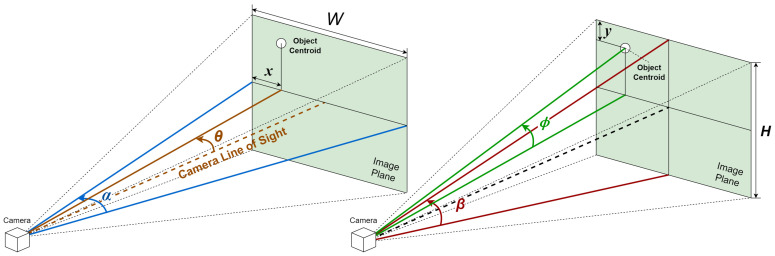
Graphical representation of azimuth and elevation calculation.

A graphical representation of the equations, illustrating the different variables, is shown in Figure 7.

To enable easier understanding and a faster response, we use the rule of thirds to divide the image plane into nine regions, and provide the location of the object in relation to the visually impaired user in terms of the region it resides in. The rule of thirds is a principle in photography and visual arts that divides the image plane into nine equal parts to help compose visual elements in a balanced and aesthetically pleasing manner. This is achieved by overlaying two equally spaced horizontal lines and two equally spaced vertical lines on the image. The intersections of these lines and the areas they define create natural points of interest and divide the space into distinct regions: upper left, upper center, upper right, middle left, center, middle right, lower left, lower center, and lower right.

**Figure 8:**
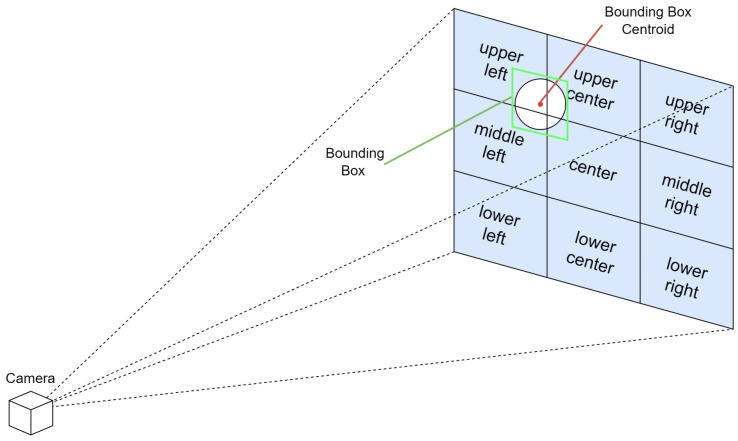
Graphical representation of region separation for intuitive object location.

In the context of assisting visually impaired users through a real-time spatial awareness system, this rule can be adapted to simplify the field of view into these nine manageable regions. By doing so, the VISA system can communicate the location of an object more intuitively. The location within the field of view is determined by the centroid of the bounding box that identifies the object in the camera’s image plane. For example, if the centroid falls within the upper left section of the grid, the VISA system would convey “upper left” to the user. Similarly, if it is in the center, the user would be informed that the object is “center”, and if in the lower right, the information provided would be “lower right”. This method allows for a straightforward and effective way of conveying spatial information, enabling visually impaired users to understand the whereabouts of objects in their immediate environment with greater ease. A graphical representation is shown in Figure 8.

#### 3.2.2. 3D Visualization

The 3D visualization module embodies the synergy between advanced computer vision techniques and user-centric design. By providing a dynamic, intuitive representation of the environment, the module plays a critical role in empowering visually impaired users to navigate and interact with their surroundings with unprecedented independence. This module not only represents a technical achievement in the field of assistive technology but also marks a significant step towards inclusive design that accommodates the needs and preferences of all users.

**Figure 9:**
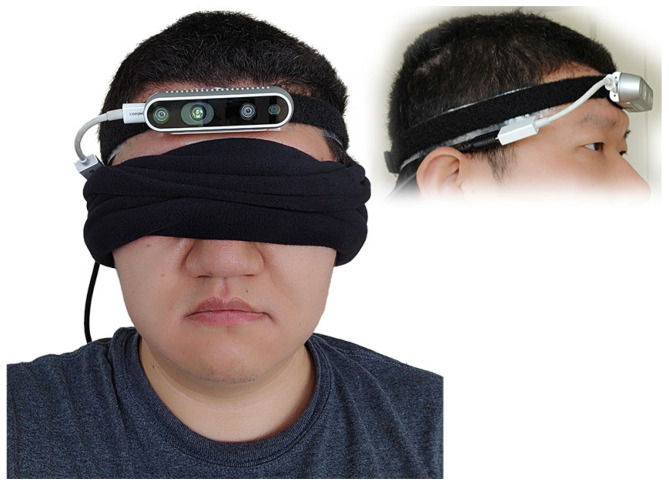
Testing configuration of the RGB-D camera worn like a headlamp.

To begin with 3D visualization, it is necessary to understand the location of the RGB-D camera, relative to the visually impaired user wearing it. We chose to wear the RGB-D camera like a headlamp, as shown in Figure 9. An assisting device to be carried by the visually impaired individual an be worn on different parts of the body, and it is important to pick the most suitable spot for the best efficiency and ease of usage. According to the review in [61], for nearly half of the assistant systems for the visually impaired they reviewed, the camera/detector was worn on the forehead or the eyes of the user. As we will show in the discussion below, this is not a coincidence.

Mounting an RGB-D camera on the forehead of a user, akin to a headlamp, offers distinct advantages for assisting visually impaired individuals in interacting with their environment. This configuration ensures the camera is positioned at a similar height and orientation to the user’s eyes, providing a field of view that closely mimics that of a sighted person. This natural alignment means the camera can capture a perspective of the world that is intuitively aligned with the user’s direction of interest, enhancing the relevance and accuracy of the information it gathers.

The placement of the camera on the forehead enables users to effortlessly scan a wide arc—up to 270 degrees—in front of them without the need to physically turn their body. This capability is particularly beneficial in crowded or confined spaces where maneuverability is limited. Users can navigate through these environments more efficiently, ensuring a smoother and safer passage.

Additionally, the intuitive ability to look up and down with the camera simplifies the process of bringing objects into the camera’s field of view for recognition. Whether it is identifying products on shelves of different heights in a grocery store or reading signage above eye level, the head-mounted camera adjusts seamlessly to the user’s natural movements, ensuring that relevant objects are easily and quickly identified without requiring manual adjustment of the device.

Turning the head to focus on a desired object or a fiducial marker centers it in the camera’s field of view, significantly simplifying the process of orientation toward a destination or item of interest. This head movement-based control mechanism allows for rapid and precise targeting, which is especially useful for detailed tasks like scanning fiducial markers for navigation within indoor spaces or selecting specific products for closer examination.

By aligning the camera’s perspective with the user’s head movements, the VISA system enhances spatial awareness and facilitates more effective interaction with the environment. This approach not only empowers visually impaired users with greater autonomy and confidence but also streamlines the process of acquiring crucial information about their surroundings, making activities like shopping, navigating complex indoor spaces, and interacting with dynamic environments more accessible and engaging.

With the information on the setup of our camera, we can come up with a 3D visualization of the objects within the field of view of the RGB-D camera. Starting from the object localization results, the average depths corresponding to the recognized objects are then mapped to the detected bounding boxes. Using the linked depth and bounding box information, a thin 3D box perpendicular to the line of sight of the camera can be created in 3D space, representing the specific physical location of the object.

**Figure 10:**
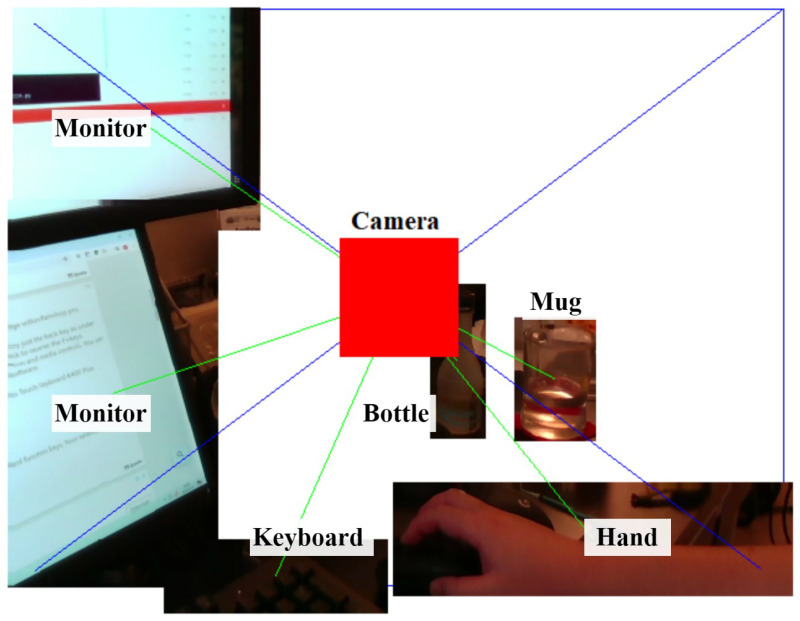
Three-dimensional visualization of environment, head-on perspective.

**Figure 11:**
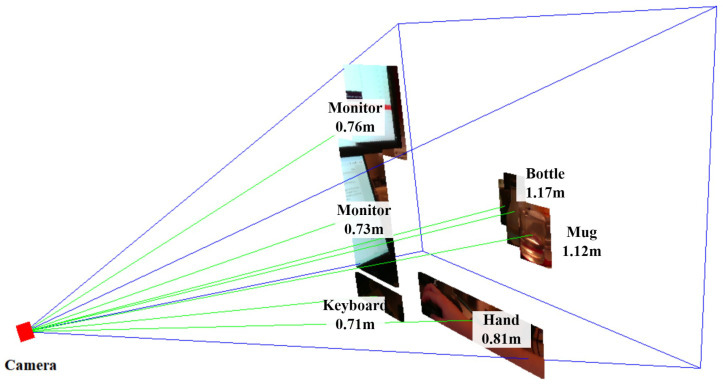
3D visualization of environment, sideways perspective.

The final step involves reconstructing the object in 3D using the color information to provide a comprehensive visualization. The pixels within the bounding box in the RGB frame are overlaid on top of the 3D box model. In this visualization, the 3D models of the objects can be interacted with by rotating the view or zooming in for more detail. Such rotation is shown in Figure 10 and Figure 11. In Figure 10, we can see the view of the RGB-D camera and the recognized objects on an image plane. However, without annotations of the distances of objects, we cannot understand the depth relationships among the objects. In Figure 11, the entire view is rotated, and we can see the different distances of the objects from the side of the camera. The rectangular pyramid with blue outlines in the two figures indicates the field of view of the RGB-D camera, with the bottom of the pyramid indicating the image plane exactly one meter away from the camera. The green lines connecting the objects and the camera indicate the azimuth, elevation, and distance of each object.

In the context of assisting visually impaired individuals, this 3D visualization could be translated into auditory feedback, providing users with an understanding of the environment around them and enhancing their spatial awareness. For instance, the VISA system could describe the size, shape, and relative position of objects, or use audio cues to indicate the direction and distance of items in a store.

**Figure 12:**
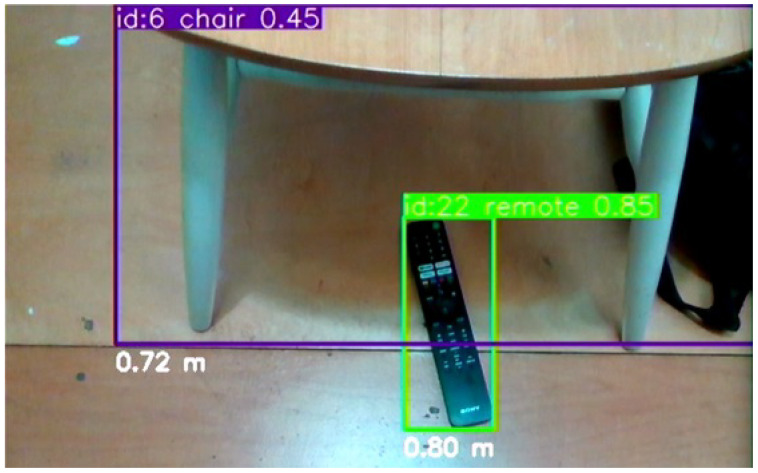
The testing setup of a TV remote on the floor next to a chair for the user to retrieve.

The VISA system was tested in an indoor environment, simulating the task of finding a specific item (a remote) on the floor. Starting with the remote within the field of view but not in the center, blindfolded users could orient their heads toward the remote within two to three seconds upon hearing the information about recognized items. Then, with the distance information provided, it was easy for the users to touch the remote in another two to three seconds. The VISA system ran at no fewer than 10 FPS for the entire duration. The setup is shown in Figure 12.

## 4. Indoor Positioning and Navigation

This section embarks on a detailed exploration of the navigation module, a cornerstone of the VISA system designed to enhance the mobility of visually impaired users within indoor environments. Central to this section is the innovative use of ArUco marker recognition integrated with depth information obtained from an RGB-D camera. This combination not only revolutionizes the way places are recognized and utilized for navigation, but also ensures safe passage by accurately detecting and avoiding obstacles. This section meticulously unpacks the technical underpinnings of the navigation system, from the initial capture of spatial data to the processing and interpretation of these data to guide users effectively. It delves into the algorithms and methodologies that enable precise and reliable ArUco marker recognition, discusses the challenges of navigating complex indoor spaces, and evaluates the VISA system’s performance in real-world scenarios. By providing a comprehensive overview of the navigation module’s development and capabilities, this section sets the stage for understanding how advanced technology can significantly improve the autonomy and safety of visually impaired individuals as they navigate through their daily lives.

### 4.1. Node Map Generation for Indoor Environment

The development of the node map for indoor navigation is based on the positioning of ArUco markers, which serve as both locational markers and data reservoirs within the built environment. This network of nodes, underpinned by fiducial marker recognition technology, is essential for guiding visually impaired users through indoor spaces by encoding spatial information in an accessible and reliable manner.

**Figure 13:**
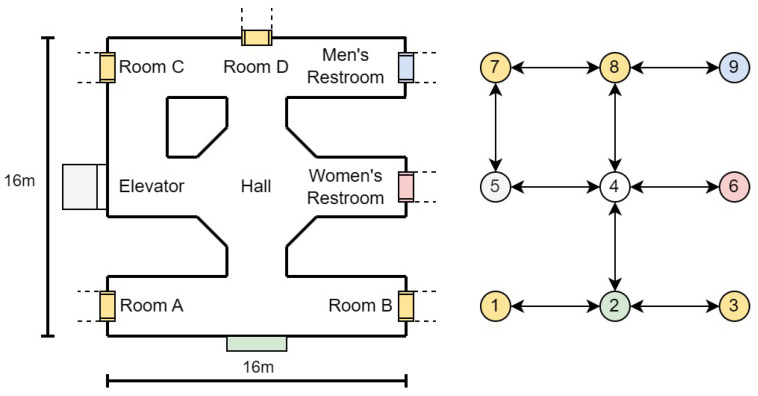
Node map generation example.

**Figure 14:**
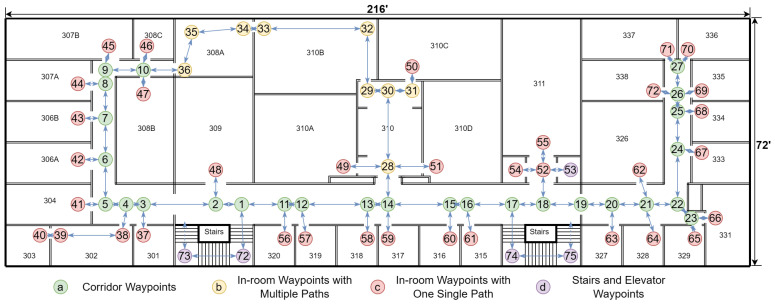
Visualization of the node map generation for Siegel Hall 3rd Floor. Room numbers are marked in black texts. The nodes are presented in the figure in a sequence.

The creation of the node map involves a survey of the indoor environment to determine the placements of necessary navigational nodes. If a drawing of the building is available, it will save time and effort in creating the node map. These nodes include decision points like corridor intersections, room entrances, and other significant landmarks that a user might need to locate or navigate around. By methodically mapping these points, we create a structured framework that reflects the physical layout and accessibility of the environment. An example of node generation is shown in Figure 13 and Table 4. A more detailed example in a real building is shown in Figure 14.

**Figure 15:**
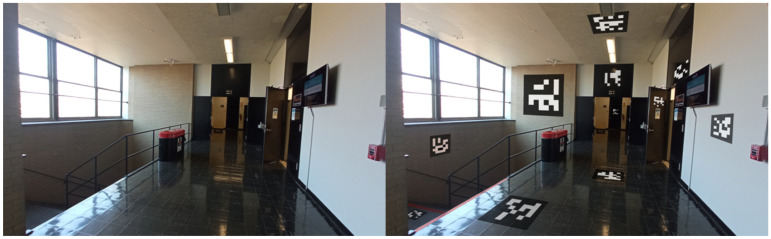
Simulated example of ArUco marker placement indoors.

Each node in the network is designated with one or more ArUco markers. These markers are matched with essential information, such as the node’s identifier and relevant metadata, including local environment descriptions. This might encompass details about adjacent rooms, directions to nearby facilities, or warnings about potential hazards. Such markers can be printed easily or even hand-drawn, and can be applied on doors, floors, or even ceilings with no permanent alteration to the indoor environment, as seen in Figure 15. When recognized by our VISA system, the ArUco markers yield precise locational data, allowing the user to orient themselves and chart a course to their desired destination.

The interaction with ArUco markers is facilitated by the assistive device’s integrated camera and image processing software, which scans and decodes the ArUco markers in real time. Upon recognition, the navigation module instantaneously updates the user’s location within the digital representation of the indoor space and proceeds to compute navigational routes. This computation takes into account the current configuration of the nodes and dynamically adjusts to any alterations within the environment, such as temporary obstructions or changes in the layout.

The visualization in the figure provided illustrates the practical implementation of such a node system. Here, the nodes are interconnected, forming a comprehensive map that not only directs the user from point to point but also informs them of their surroundings. This approach facilitates an intuitive understanding of the space and enhances the user’s ability to navigate it independently.

The integration of the node map with other system components, specifically the object recognition and localization module, is a pivotal aspect of the design. The object recognition and localization module informs the user about immediate obstacles and items of interest. The navigation module ties these elements together, providing the user with both micro-level detail and macro-level orientation within the indoor environment.

In summary, the node map is a fundamental element of our indoor navigation module, embodying the synergy of ArUco marker technology, spatial mapping, and user-centric design to empower visually impaired individuals with enhanced mobility and spatial awareness. Its implementation within the module showcases an innovative approach to indoor navigation, advancing the state of assistive technologies for visually impaired users.

### 4.2. ArUco Marker Recognition

This section delves into the ArUco marker recognition module, a pivotal component of the indoor navigation module designed to enhance the autonomy of visually impaired individuals. ArUco markers serve as effective node designators within the indoor environment, providing users with precise locations of places and facilitating seamless navigation. Its implementation leverages advanced image processing techniques to detect and decode ArUco markers in real time, employing the camera system to capture visual inputs. Upon recognizing an ArUco marker, the VISA system matches relevant location data, enabling the dynamic generation of vocal guidance for the user. This method not only ensures accurate marker identification but also integrates smoothly with the VISA system’s depth-sensing capabilities, offering a comprehensive solution for obstacle avoidance and pathfinding. The ArUco marker recognition process is optimized for efficiency, ensuring minimal latency and high accuracy in various lighting conditions, thereby empowering visually impaired users with a reliable means of indoor navigation.

The maximum distance at which an ArUco marker can be effectively recognized is a critical factor for indoor navigation. An empirical equation exists for scanning distance using cameras on common smartphones [62], d=250l/R. Here, *d* is the maximum scanning distance, *l* is the side length of the ArUco marker in meters, and *R* is the number of rows or columns in the ArUco marker.

As the equation suggests, we can trade information for recognizability. By using a code with fewer rows and columns like ArUco markers, we can reduce the number of rows and columns to 6, thus increasing the maximum scanning distance to more than 10 m assuming *l* = 0.3 m. This distance calculation allows the VISA system to dynamically adjust the granularity of navigation instructions based on the user’s proximity to the next node. Such dynamic feedback is crucial for facilitating smooth and intuitive navigation, significantly enhancing the spatial awareness of visually impaired individuals within indoor environments.

### 4.3. Indoor Positioning Using ArUco Marker Recognition

In the domain of indoor navigation, the strategic deployment of ArUco markers integrated with depth sensing technology has emerged as a cornerstone for developing indoor positioning systems. This section delves into the innovative application of ArUco markers for indoor positioning, an essential component of the VISA system for visually impaired individuals. Through a detailed analysis of the underlying graph structure and algorithms, we illustrate how the VISA system accurately determines the camera’s (and thus the user’s) position and orientation within an indoor environment.

The basis of our positioning system is encapsulated in a graph structure that represents the spatial layout of indoor environments using ArUco markers. Each ArUco marker within this graph is associated with a tuple comprising the marker’s coordinates and a normalized vector indicating the marker’s orientation. For instance, in an example graph, the markers are identified by integers (e.g., 4, 8, and 996), with each node’s spatial coordinates and orientation vectors provided. This is shown in Table 5.

The coordinates represent the physical location of each ArUco marker in the indoor space, whereas the direction vectors indicate the orientation of the ArUco markers. This orientation is required to deduce the camera’s direction in the indoor environment.

The core of our indoor positioning system lies in the dynamic recognition of ArUco markers and the interpretation of their spatial information to ascertain the camera’s location and facing direction. Recognized markers are logged into a list, which aggregates the ID, the rotation matrix based on the angle of incidence, and the distance from the camera for each detected marker.

The coordinates of the user can be calculated, based on the incidence angles of the recognized markers and their distances. Furthermore, the horizontal direction of each marker relative to the camera is computed using a function that translates the centroid’s horizontal coordinate of a marker into an angle of incidence based on the camera’s field of view (FOV) and resolution. The calculated angle aids in determining the marker’s position relative to the camera’s central axis, thereby facilitating a nuanced understanding of the camera’s orientation within the space. The calculation of the user’s coordinates is shown in the equation below:

(1) $${\vec{C}}_{\mathrm{user}}={\vec{V}}_{m}+dR\left(\theta \right){\vec{V}}_{d},$$

where ${\vec{C}}_{\mathrm{user}}$ is the vector determined by the coordinates of the user (x,y), ${\vec{V}}_{m}$ is the vector determined by the coordinates of the marker, *d* is the measured distance, ${\vec{V}}_{d}$ is a normalized vector representing the direction that the marker is facing, and R(θ) is the rotation matrix based on the angle of incidence θ, $R\left(\theta \right)=\left[\begin{array}{cc} \cos\theta & -\sin\theta \\ \sin\theta & \cos\theta \end{array}\right]$.

For a total of *n* ArUco markers within the field of view, we have the equation below:

(2) $$\bar{{\vec{C}}_{\mathrm{user}}}=\frac{1}{n}\sum_{i=1}^{n}\left({\vec{V}}_{mi}+d_{i}R\left(\theta_{i}\right){\vec{V}}_{Di}\right)$$

Similarly, we can calculate the normalized vector representing the user’s direction, literally where the user is facing, using the equation below, where $R(\theta^{\prime })$ is the user’s direction rotation matrix based on the angle of the marker relative to the user’s direction $\theta^{\prime }$, $R\left(\theta^{\prime }\right)=\left[\begin{array}{cc} \cos\theta^{\prime } & -\sin\theta^{\prime }\\ \sin\theta^{\prime } & \cos\theta^{\prime } \end{array}\right]$:

(3) $${\vec{V}}_{d,\mathrm{user}}=-R\left(\theta \right)R\left(\theta^{\prime }\right){\vec{V}}_{d}$$

**Figure 16:**
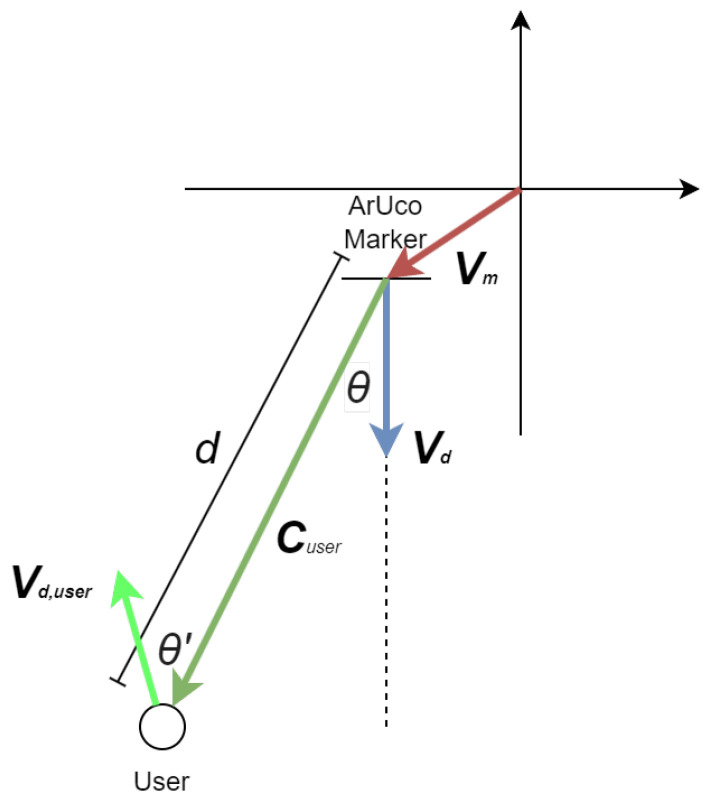
Visualization of coordinates calculation for visually impaired users.

A visualization of the calculations is shown in Figure 16.

As can be seen in the equations above, the depth information and the directional data of recognized markers are used to compute the user’s estimated position and orientation. Notably, the function incorporates adjustments for the camera’s position based on the markers’ orientation and distance, exemplifying a reverse-engineering approach to infer the camera’s perspective from the markers’ spatial data. Finally, it should be noted that the positioning accuracy increases with a reduction of in incidence angle, so it is advantageous to place more ArUco markers to reduce the incidence angles of detected markers. This methodology underscores the adaptability of the VISA system to varying indoor environments, a testament to its potential as a reliable navigational aid.

**Figure 17:**
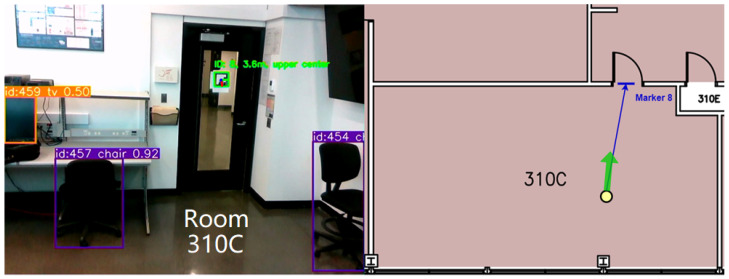
Indoor positioning using one ArUco marker in Siegel Hall 310C. Green arrow indicates direction user is facing.

The algorithm’s efficacy is demonstrated through its ability to average multiple marker detections, thereby mitigating the impact of potential outliers and ensuring a smooth and accurate positioning experience. This feature is particularly beneficial in densely populated indoor settings, where markers are abundant and viewpoints may vary significantly. A real example is shown in Figure 17. In this test, the size of the ArUco markers is 190 mm × 190 mm. It can be seen that the ArUco marker with ID 8 can be recognized at a distance of 3.5 m under indoor lighting conditions when the user is walking at a slow pace. With larger ArUco markers, our ArUco marker recognition module can reliably recognize the markers at a longer distance, making the ArUco markers suitable for indoor positioning in larger buildings with fewer markers. Also, it can be seen that the object recognition module and the ArUco marker recognition module are running at the same time.

In summary, the indoor positioning system leveraging ArUco markers presents an efficient and cost-effective solution to the challenges of real-time navigation for visually impaired individuals. By intricately analyzing the spatial information encoded in ArUco markers, the VISA system offers precise and responsive feedback on the user’s location and orientation, thereby enhancing their spatial awareness and mobility within indoor environments.

### 4.4. Path Planning Based on Node Map

In the context of enhancing indoor navigation for visually impaired users, path planning plays a pivotal role in ensuring seamless and safe movement through environments. Leveraging the foundational groundwork laid by the node map generated in Section 4.1, this section delves into the methodologies and algorithms pivotal for crafting dynamic and efficient navigation paths.

The core of the path planning mechanism involves dynamically calculating the most efficient route from the user’s current location to their desired destination. This is achieved by integrating the precise location data obtained from ArUco marker recognition, as outlined in Section 4.2, with the detailed node map. The path planning algorithm meticulously analyzes the spatial layout, identifying optimal pathways while considering the shortest physical distance.

To navigate the complexities of indoor environments, the VISA system employs Dijkstra’s algorithm [63]. This algorithm is renowned for its efficiency in finding the shortest path between points in a graph, making it ideal for real-time navigation purposes. Its implementation takes into account the physical distance, ensuring the selected path is the shortest for visually impaired users. The procedures of the Dijkstra’s path calculation algorithm are given below.

1. Initialization:
  Assign an initial distance of infinity to all nodes, except the starting node, which is set to 0. Mark the starting node as the current node.
2. Unvisited Set:
  Mark all nodes as unvisited. Create a set containing all unvisited nodes, called the “unvisited set”.
3. Destination Check:
  Check if the destination node has been visited.
  If yes, proceed to the end and retrace the steps to find the optimal path.
  If no, continue to the next step.
4. Select the Current Node:
  Select the unvisited node with the smallest distance as the current node.
5. Neighbor Check:
  Inspect the neighbors of the current node.
6. Distance Update:
  For each neighbor, check if the path through the current node offers a shorter distance.
  If yes, update the distance for that neighbor.
7. Neighbor Completion Check:
  Check if all neighbors of the current node have been visited.
  If no, repeat the neighbor check for the next neighbor.
  If yes, mark the current node as visited and remove it from the unvisited set.
8. Loop:
  Repeat steps 4 through 8 until the destination node is visited.
9. Path Retrace:
  Once the destination node is visited, retrace the steps to determine the optimal path from the start to the destination.
10. End:
  Conclude the process. The shortest path and its distance are now identified.

An essential aspect of the path planning process is its adaptability to real-time changes within the environment. The indoor spaces traversed by the visually impaired user can be dynamic, with the potential for alterations in layout due to moved furniture or the presence of temporary obstacles like people or carts. The VISA system continuously monitors the environment for changes, adjusting the proposed navigation path as necessary to maintain its viability and safety. This is further discussed in Section 4.3.

The efficiency and reliability of the proposed navigation paths are assessed through comprehensive simulations and real-world testing. These evaluations focus on metrics such as navigation time, the accuracy of the path with respect to the destination, and user feedback regarding the ease of following the suggested path. This iterative evaluation process ensures continuous improvement of the path planning algorithm, aiming to enhance the overall user experience in navigating indoor environments.

### 4.5. Obstacle Avoidance Based on Depth Information

To further enhance the navigation capabilities of visually impaired individuals in indoor environments, obstacle avoidance functionality needs to be implemented in our VISA system. This section describes the integration of object recognition and localization, utilizing YOLOv8s for recognition and using depth information from depth cameras for localization, as detailed in Section 3, to identify and circumnavigate obstacles effectively.

The VISA system’s obstacle avoidance mechanism operates on two fronts. Firstly, it uses the results of object recognition and localization to identify obstacles within the user’s path, leveraging the depth information to gauge the distance and dimensions of these obstacles accurately. This allows for the dynamic adjustment of the navigation path to avoid these obstacles, ensuring safe passage for the user.

**Figure 18:**
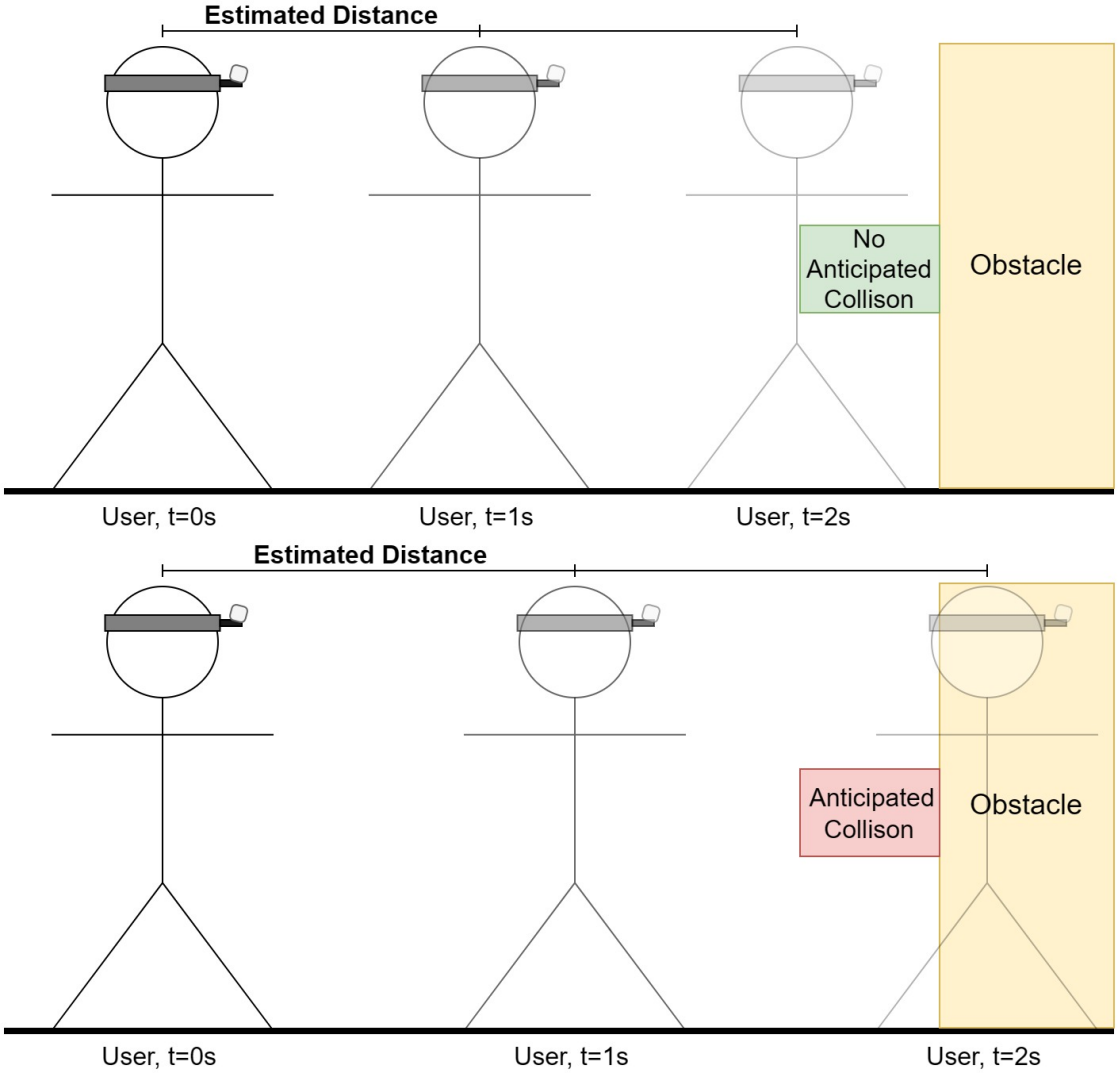
Collision forecast based on projected location for different closure rates.

Secondly, the VISA system employs a direct analysis of the depth frame, focusing on a grid of sampling points to monitor the space in front of the user. By evaluating the distance data from these points, the VISA system can detect sudden changes in depth that signify the presence of an obstacle. When a sampling point indicates a distance shorter than a safety threshold, the VISA system issues a warning to the user, enabling them to stop or change direction. Instead of using a predetermined threshold, the threshold is calculated based on the rate of closure of pixels in the region of interest on the depth image. The region of interest is chosen so that regions that are not in the predicted path will be ignored. The regions chosen are the top middle, the center, and the lower center regions in Figure 8. The closure rate can be determined using Equation (4). Examples of different closure rates are shown in Figure 18.

(4) $$R=\frac{\left(\frac{1}{W\times H}\sum_{i=1}^{W\times H}D_{i,\mathrm{previous}}\right)-\left(\frac{1}{W\times H}\sum_{i=1}^{W\times H}D_{i,\mathrm{current}}\right)}{\Delta t}$$

The variables in the equation are defined as follows:

1. *R* is the rate of closure, representing the speed at which the observer is moving towards or away from the object or scene in focus, measured in meters per second.
2. $D_{i,\mathrm{current}}$ represents the depth of the *i*th pixel in the current depth frame.
3. $D_{i,\mathrm{previous}}$ represents the depth of the *i*th pixel in the previous depth frame.
4. *W* and *H* are the horizontal and vertical resolutions of the region of interest, respectively, indicating the number of pixels along each dimension.
5. $\sum_{i=1}^{W\times H}D_{i,\mathrm{frame}}$ is the summation of depths of all pixels in a given region of interest, where *frame* can be either *current* or *previous*.
6. Δt is the change in time between the capture of the current and previous depth frames, typically measured in seconds.

Following the calculation of the closure rate, we can calculate the safety threshold. To calculate the safety threshold distance based on the time to collision (TtC), we use the concept that the time to collision can be determined by dividing the current distance to the object by the rate of closure, assuming the rate of closure remains constant. This concept allows us to establish a safety threshold by determining how much distance is considered safe given a certain amount of time before a potential collision occurs.

The equation to calculate the Time to Collision (TtC) and the safety threshold distance is given by TtC = $D_{\mathrm{current}}$/*R*. Here, $D_{\mathrm{current}}$ is the current distance to the object (calculated as the average depth of the pixels in the current frame), and *R* is the rate of closure. To establish a safety threshold, we decide on a minimum safe TtC (denoted as $TtC_{\mathrm{safe}}$ ), and rearrange the equation to solve for the safety threshold distance: $D_{\mathrm{safe}}$ = *R* × $TtC_{\mathrm{safe}}$. The variables in the safety threshold distance equation are defined as follows:

1. $D_{\mathrm{current}}$ represents the current distance to the object or scene in focus. It is calculated as the average depth of all pixels in the current depth frame, providing an estimate of how far the observer is from a point of interest or obstacle.
2. *R* is the rate of closure between the observer and the object or scene. It quantifies the speed at which the distance to the object is decreasing (or increasing if moving away), measured in meters per second. A positive rate indicates that the observer is moving towards the object, while a negative rate suggests they are moving away.
3. TtC stands for Time to Collision. This variable estimates the amount of time remaining before a collision occurs with the object or scene, assuming the current rate of closure *R* remains constant. It is calculated by dividing the current distance $D_{\mathrm{current}}$ by the rate of closure *R*.
4. ${\mathrm{TtC}}_{\mathrm{safe}}$ is the predefined minimum safe Time to Collision. This value represents the desired buffer time that should be maintained to prevent collisions, allowing sufficient time for corrective actions to be taken. It is a safety parameter set based on the specific requirements of the navigation module or user preferences.
5. $D_{\mathrm{safe}}$ denotes the safety threshold distance, which is the critical distance that must be maintained from an object or scene to ensure safety, given the predefined ${\mathrm{TtC}}_{\mathrm{safe}}$. It is determined by multiplying the rate of closure *R* by the minimum safe Time to Collision ${\mathrm{TtC}}_{\mathrm{safe}}$, yielding the distance at which preventive or corrective measures should be initiated to avoid a potential collision.

Through the combination of these strategies, the VISA system provides a robust solution for obstacle detection and avoidance, significantly enhancing the safety and efficiency of indoor navigation for visually impaired individuals. This approach not only leverages the advanced capabilities of AI and depth sensing technologies but also emphasizes the importance of real-time adaptability and user feedback in creating a comprehensive navigational aid with obstacle avoidance functionality.

## 5. Human–Machine Interface for Visually Impaired Users

In the realm of assistive technologies for the visually impaired, effective HMI is paramount. The need for intuitive, responsive, and accessible communication channels cannot be overstated, as they directly impact the user’s ability to interact with the environment, perform tasks, and engage in different activities. Thus, we provide a detailed description in this section, dissecting the various components that constitute the VISA system’s human–machine interface, focusing on both input and output mechanisms that cater to the specific needs of visually impaired users.

We commence with an in-depth analysis of the text-to-speech module, which serves as the auditory channel for conveying essential information and feedback to the user. Following this, we examine the speech-to-text module, highlighting its role in interpreting user commands and enabling a natural, voice-driven interaction with the VISA system. The discourse then extends to character and object recognition using Google Lens, illustrating how advanced visual recognition technologies can empower users to understand and interact with their surroundings more effectively.

Through the exploration of these key areas, this section aims to underscore the importance of a robust, user-centered human–machine interface in the development of assistive technologies. In the last part of this section, we compare our VISA system with existing items and systems to assist the visually impaired, in terms of practicality and functionality.

### 5.1. Text-to-Speech Module

The text-to-speech (TTS) module represents a cornerstone of the interactive system designed to empower visually impaired individuals by facilitating the translation of textual information into audible speech. This module plays a pivotal role in enhancing the autonomy and navigational capabilities of the user, by providing real-time, audible feedback about their immediate environment, recognized objects, and navigation cues. The implementation of the TTS module leverages the pyttsx3 library, a cross-platform tool that interfaces with native TTS engines on Windows, macOS, and Linux, offering a high degree of compatibility and customization.

The pyttsx3 library was chosen for its robustness, its ease of integration, and the quality of its speech output. The initialization of the TTS engine is straightforward, facilitating rapid deployment and real-time interaction with the user. The engine is configured to operate in a separate threading model to avoid blocking the main execution thread, thus ensuring that speech output does not interfere with the continuous processing of sensory data and object recognition tasks.

The TTS module is used for multiple tasks of the VISA system, including:

1. Announcing detected objects and their locations relative to the user.
2. Reading ArUco markers identified in the environment, providing contextual information and navigation assistance.
3. Issuing warnings for obstacle avoidance.
4. Reading text from the recognition results of Google Lens.
5. Interacting with user voice commands or reciting them for confirmation.

To optimize the user experience, the TTS module was customized in several key aspects. The speech rate and volume were adjusted to ensure clarity and audibility, considering the diverse environments in which the VISA system may be used. Furthermore, the selection of voices was tailored to cater to user preferences and accessibility requirements, enhancing the naturalness and engagement of the interaction.

The integration of the TTS module within the broader system architecture is seamless, with APIs facilitating the dynamic generation of speech output based on real-time data from the object recognition and localization modules, as well as user inputs processed through the speech-to-text module. This integration underscores the modular design of the VISA system, where the TTS module functions as an essential interface for human–machine communication.

To summarize, the text-to-speech module serves as a must-have component of the VISA system, embodying the commitment to providing visually impaired users with a comprehensive, intuitive, and accessible navigational aid. Through careful selection of technologies, customization to meet user needs, and seamless integration with the VISA system’s architecture, the TTS module significantly contributes to the overarching goal of enhancing the autonomy and quality of life of visually impaired individuals.

#### Threading in the Text-to-Speech Module

In the implementation of the text-to-speech (TTS) module within our VISA system, threading is a key technique to enhance the VISA system’s responsiveness and usability, particularly for visually impaired users requiring real-time auditory feedback. The utilization of the threading library in Python facilitates the execution of multiple operations concurrently, thereby ensuring that the VISA system’s main computational processes remain uninterrupted by TTS operations.

The primary motivation behind employing threading for the TTS module stems from the necessity to maintain seamless system performance while executing potentially blocking operations such as speech synthesis. Given the VISA system’s objective to provide instant feedback based on real-time environmental data and user interactions, it is imperative that these feedback mechanisms do not hinder the VISA system’s core functionalities, including object detection, navigation, and user command processing.

**Figure 19:**
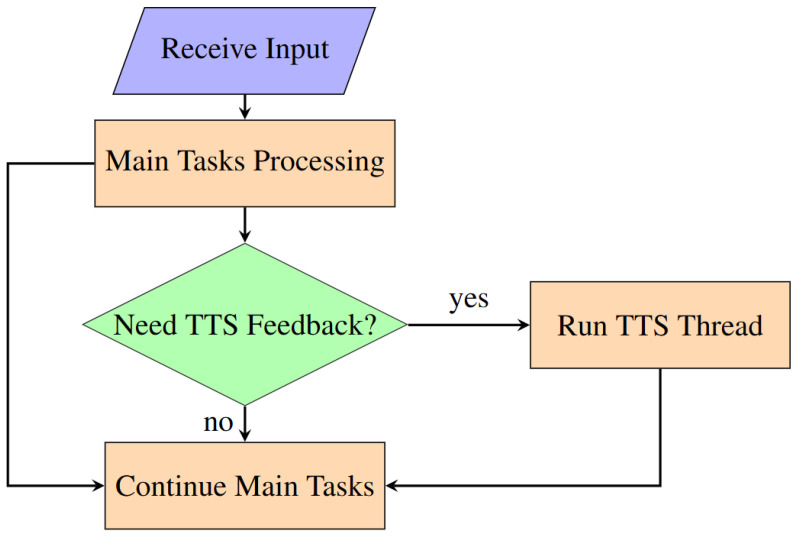
Flowchart for threading in VISA system program.

In the system code, threading is utilized to initiate speech synthesis tasks in parallel with the main application processes. This is achieved by encapsulating the TTS functionality within a separate thread, effectively isolating it from the primary execution flow. The specific implementation involves the creation of a speak text thread, which serves as the entry point for the TTS operations. Upon the need for a TTS module, the thread is dedicated to executing the speech synthesis task, thereby allowing the VISA system to continue its operation without waiting for the speech output to complete. The use of the speech thread-running flag ensures that only one instance of speech synthesis is active at any given time, preventing overlapping speech outputs and managing the queue of speech requests effectively. A flowchart for threading in our VISA system is shown in Figure 19.

The adoption of threading in the TTS module introduces several benefits:

Non-blocking Operations: By offloading speech synthesis to a separate thread, the VISA system can continue to process sensory inputs, detect objects, and respond to user commands without delay, ensuring a fluid user experience.

Improved Responsiveness: The VISA system can provide immediate auditory feedback to user actions or environmental changes, a crucial aspect for navigation and interaction in real-time scenarios.

Enhanced System Stability: Isolating the TTS operations in a separate thread reduces the risk of system slowdowns or crashes that could result from the synchronous execution of resource-intensive tasks.

The strategic use of threading in the TTS module significantly contributes to the overall performance and user experience of the VISA system. By enabling the concurrent execution of speech synthesis alongside critical system processes, threading ensures that the VISA system remains responsive and effective in providing real-time assistance to visually impaired users.

### 5.2. Speech-to-Text Module

The speech-to-text (STT) module constitutes an essential component of the human–machine interface within our VISA system designed for visually impaired individuals. This module facilitates an intuitive and efficient means for users to interact with the VISA system through voice commands, significantly enhancing the VISA system’s accessibility and usability. Leveraging advanced speech recognition technologies, the STT module converts spoken language into text, enabling the VISA system to understand and act upon user commands in real time.

The STT functionality is implemented using the speech recognition library, known for its versatility and support for multiple speech recognition services, including Google Speech Recognition. This choice aligns with the VISA system’s need for reliable and accurate speech-to-text conversion, ensuring that user commands are interpreted correctly under various conditions.

**Figure 20:**
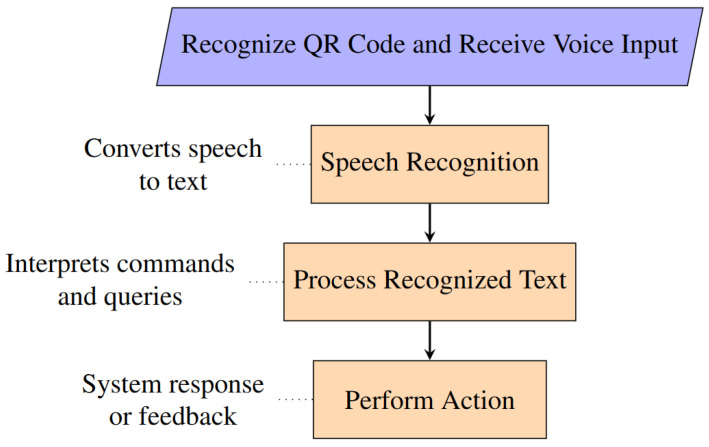
Flowchart for speech-to-text module.

The STT module can be invoked upon recognizing a QR code in the field of view of the camera. The user just needs to place the QR code close to the RGB-D camera to issue commands, with no need for other I/O devices. Upon the start of STT service, the audio is captured and forwarded to the speech recognition service, which processes the audio and returns the corresponding textual representation. This process is encapsulated within the STT function, illustrating the module’s operation, as shown in Figure 20.

The STT module is seamlessly integrated into the broader system architecture, enabling users to issue voice commands that control various system functionalities, such as navigation commands, requests for information about nearby objects, or commands to repeat the last spoken feedback. The VISA system’s ability to interpret these commands accurately and provide the appropriate feedback or action is paramount to its effectiveness as an assistive tool.

The following commands related to object recognition can be issued by the user:

1. List: The VISA system lists all recognized objects in the field of view. Example: “Detected objects are: chair, remote”.
2. Look for [Object Class]: The VISA system looks for a specific class of the item in the field of view, and announces its location upon recognition. A [Looking] flag is set, indicating the VISA system is now in item search mode. Reset all other flags. Example: “Remote center, zero point eight meters”.
3. Locate: The VISA system looks for ArUco markers in the field of view and announces its corresponding place upon recognition. Example: “Entrance, middle center, zero point six meters”.
4. Go to [Node Name]: The VISA system uses Dijkstra’s Algorithm to determine the path to the place announced by the user, and provides instructions based on results from the positioning module. A [Navigating] flag is set, indicating the VISA system is now in navigation mode. The system now automatically announces AruCo markers it recognizes, providing the user with positional information. Reset all other flags. Example: “Turn left ninety degrees for SPAM shelf, one meters”.
5. Stop: Reset all flags, exiting from looking mode or navigation mode.
6. Upload: Upload the current color frame to Google Lens and read the results.
7. Upload Recognized [Object Class]: The VISA system will upload the images within bounding boxes corresponding to the said object class. Read the results.

Implementing an effective STT module within the VISA system presented several challenges, primarily related to achieving high accuracy and responsiveness under varying acoustic environments. Background noise and variations in speech patterns can significantly affect the module’s performance. To mitigate these issues, the VISA system employs noise reduction techniques to enhance recognition accuracy.

Moreover, the reliance on external speech recognition services introduces concerns regarding latency and availability. The VISA system addresses these by optimizing the audio capture and transmission process, and by incorporating fallback mechanisms to ensure continued functionality even when the primary service is unavailable. For example, a timeout is implemented in our VISA system, preventing constant waiting for speech in case of erroneous invoking of the STT module.

To summarize, the speech-to-text module provides a natural and accessible interface for visually impaired users to interact with our VISA system. Through the careful selection of speech recognition technologies, the module contributes to the VISA system’s overall goal of enhancing the autonomy and mobility of visually impaired individuals.

## 6. Case Study: Grocery Shopping

**Figure 21:**
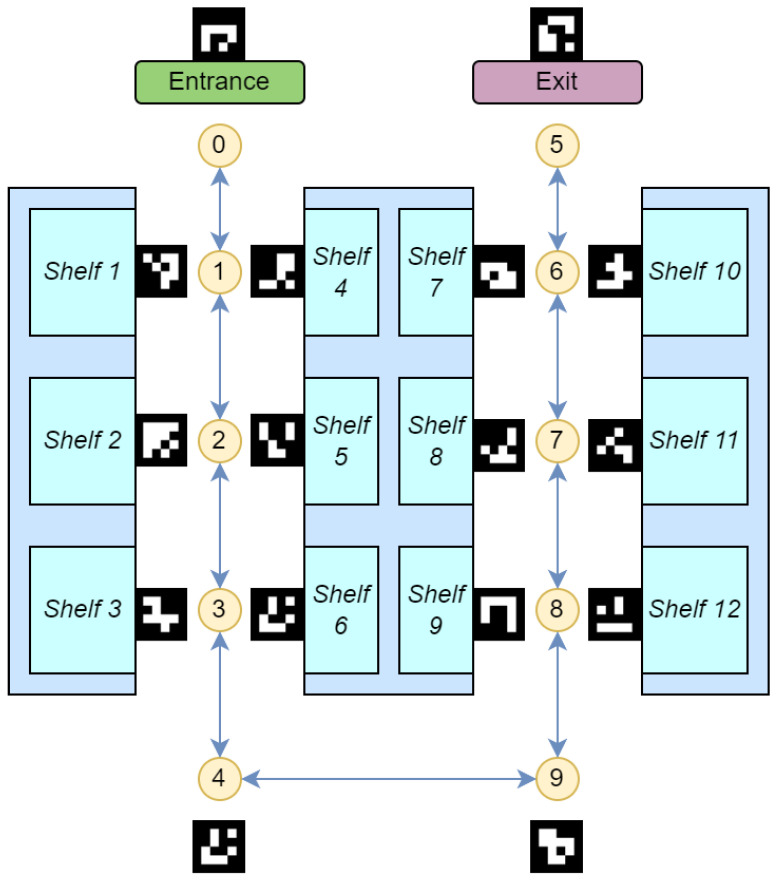
Grocery store simulation environment for navigation and product fetching. The nodes and the shelves are numbered in a sequence.

**Figure 22:**
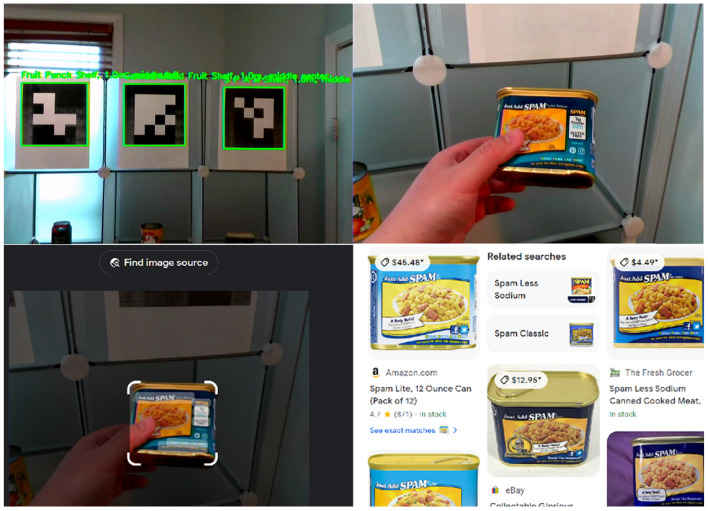
Example of shelf recognition using ArUco markers, picking up merchandise, and using Google Lens to recognize the merchandise.

A comprehensive test in a simulated grocery store was conducted with satisfactory results. In the test, the individual can utilize the vocal cues provided by the VISA system, navigate in the simulated environment, pick up the desired items from the correct shelf, confirm selection, and proceed to the checkout/exit. An example layout of part of a grocery store is shown in Figure 21. An example of shelf recognition using ArUco markers, picking up merchandise, and using Google Lens to recognize the merchandise in this store is shown in Figure 22. A list is provided below, looking into the different aspects of using the VISA system to assist in grocery shopping.

1. Challenges in Grocery Shopping
  Visually impaired individuals face significant challenges in grocery shopping, such as navigating store layouts, identifying products, and accessing product details. Existing solutions often focus narrowly on either navigation or product identification, requiring costly infrastructure like RFID tags. Few systems address both functionalities comprehensively [64].
2. ArUco Markers for Navigation
  ArUco markers provide a cost-effective and flexible solution for store navigation. Placed strategically throughout the store, they enable the creation of a node map that integrates with the VISA system. These markers guide users dynamically, offering positional updates and optimized route calculations.
3. Object Recognition and Localization
  The VISA system leverages YOLOv8 for real-time object recognition, enabling users to identify products and obstacles within their environment. Depth data enhance this capability by providing spatial localization of objects. For detailed product identification, Google Lens delivers specific insights, such as nutritional information and pricing.
4. Obstacle Avoidance and Shelf Recognition
  The VISA system employs depth-based algorithms for dynamic obstacle avoidance, ensuring safe navigation in crowded environments. By recognizing shelves and their contents through ArUco markers and YOLOv8, the system facilitates efficient product retrieval. Google Lens enhances the user experience by reading detailed product labels and logos.
5. Human–Machine Interface (HMI)
  The system’s HMI incorporates speech-to-text (STT) and text-to-speech (TTS) technologies. Users can issue voice commands to navigate, identify products, and interact with the system. TTS provides real-time feedback, confirming user actions and delivering navigational guidance. This seamless interaction reduces cognitive load, making the shopping experience intuitive and accessible.
6. System Integration and Testing
  The VISA system integrates its modules—navigation, object recognition, obstacle avoidance, and HMI—into a cohesive framework. Testing in a simulated grocery store demonstrated the system’s effectiveness. Users successfully navigated aisles, identified products using ArUco markers and Google Lens, and completed shopping tasks independently.
7. Conclusions
  The VISA system redefines accessibility for visually impaired individuals in grocery shopping. By addressing navigation, product identification, and human–machine interaction holistically, it promotes independence, inclusivity, and convenience, making daily tasks more achievable.

## 7. System Comparisons

**Figure 23:**
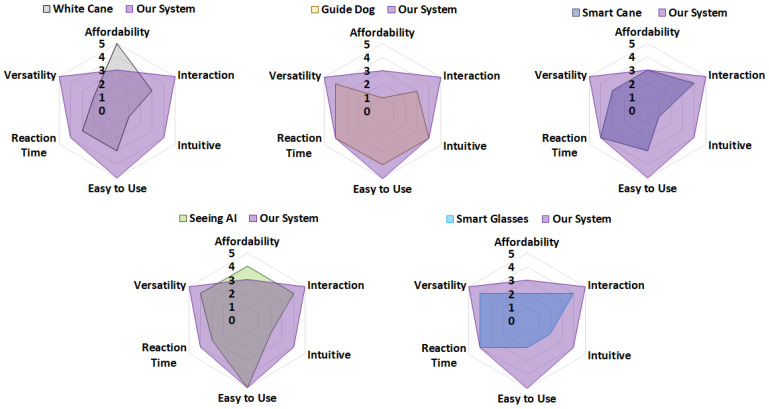
Radar chart comparing the VISA system’s practicality with that of other techniques.

**Figure 24:**
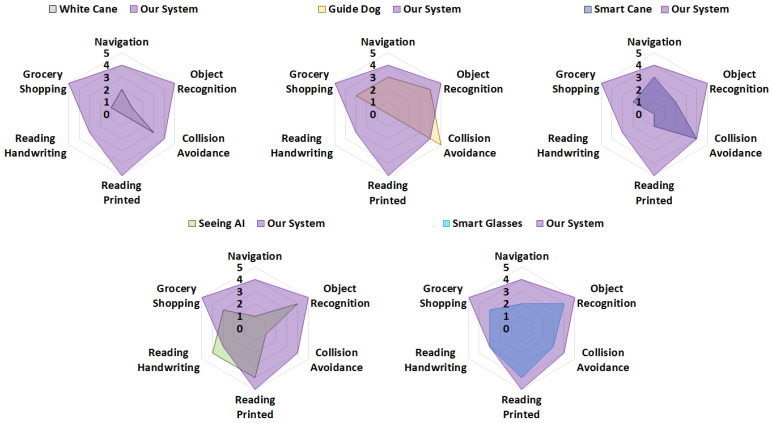
Radar chart comparing the VISA system’s functionality with that of other techniques.

In this section, we perform a conceptual comparison of the practicality and functionality of the VISA system with other techniques. The compared techniques include white canes, guide dogs, typical smart canes with ultrasonic or other forms of collision avoidance [65,66,67], the Seeing AI smartphone app developed by Microsoft [68], and typical smart glasses with object recognition [69,70,71]. The Seeing AI’s app’s counterpart, Lookout—Assisted Vision developed by Google, has similar performance [72,73]. The results are shown in Figure 23 for a practicality comparison and in Figure 24 for a functionality comparison.

For the practicality comparison, we scored each system or technique from 0 to 5 based on six attributes, namely affordability, interaction, intuitiveness, ease of use, reaction time, and versatility. Similarly, for the functionality comparison, we scored each system or technique from 0 to 5 based on six attributes, namely navigation, object recognition, collision avoidance, reading printed texts, reading handwriting, and grocery shopping. A higher score indicates better performance in the specific attribute. For instance, white canes receive an affordability score of 5, owing to their simple construction and low cost, whereas guide dogs are assigned an affordability score of 1, reflecting their accessibility to only a small group of individuals due to their high cost.

The proposed VISA system offers a balanced and practical solution for visually impaired individuals, addressing the limitations of conventional techniques across both practicality and functionality metrics. As can be seen in the figures, for practicality, our VISA system sits behind white canes and APPs only in terms of affordability; for functionality, our VISA system is superior to all other techniques, with only guide dogs being equal in terms of collision avoidance, and APPs in terms of reading texts.

In terms of practicality, the VISA system demonstrates a well-rounded balance when compared to conventional solutions such as white canes, guide dogs, smart canes, Seeing AI, and smart glasses. As shown in the radar charts, VISA excels in interaction, ease of use, and versatility, making it more accessible and user-friendly than many other alternatives. Traditional tools like white canes are affordable but lack versatility and intuitiveness, while guide dogs offer strong interaction and intuitiveness but are expensive and require significant training resources. Advanced electronics like smart canes and glasses often provide better interaction and versatility, but neither is intuitive to use. Smart glasses have the added disadvantage of a high price tag. To summarize, the VISA system strikes a strong balance, offering top performance in all five of the other fields while remaining affordable for daily use.

From a functionality perspective, the VISA system competes effectively with conventional solutions, particularly in grocery shopping, navigation, and object recognition. It outperforms all other systems in these areas, and only trails slightly behind AI-powered systems such as Seeing AI in tasks requiring handwriting recognition or complex scene interpretation. Also, for collision avoidance, which is one of the key tasks in assisting the visually impaired and has been extensively researched, our VISA system still outperforms all the other systems except for guide dogs. To summarize, the VISA system effectively bridges the gap in conventional single-task systems by providing robust functionality for common daily tasks like grocery shopping, navigating indoor spaces, and reading printed text.

## 8. Conclusions

This paper introduces the VISA system, a holistic solution designed to assist visually impaired users with various indoor activities using a multi-level approach. Most existing systems and tools in this domain are single-task-focused and unable to address the diverse tasks faced by visually impaired individuals in complex indoor environments. Consequently, a holistic solution capable of handling multiple tasks can significantly enhance the independence of visually impaired users in such settings. By leveraging recent advancements in computer vision, deep learning, embedded systems, and edge computing, we have successfully developed the VISA system to fulfill the key objectives of a holistic solution.

In summary, the VISA system serves as a comprehensive aid for visually impaired users, providing a suite of functionalities to assist them in their daily activities. By detecting and recognizing common objects within the field of view of the RGB-D camera, the VISA system provides users with a list of nearby objects without requiring physical contact. By conveying direction and distance information of recognized objects, our VISA system enables the user to locate and retrieve items efficiently. By providing navigational cues and auditory warnings, our VISA system helps users reach their indoor destination and avoid obstacles with minimal effort. Moreover, using Google Lens allows users to accurately identify items and read a variety of textual media, such as product labels, handwritten notes, and printed documents. Integrating all the aforementioned functionalities and utilizing their generated information, we deliver holistic assistance that empowers visually impaired users to accomplish a broader scope of tasks with increased efficiency and safety. With experimental results from tests in different environments simulating real-world scenarios, we conclude that our VISA system is easy to use and can assist visually impaired users in nearly all aspects of their daily life, particularly in finding objects, navigating indoor spaces while avoiding obstacles, discerning items of interest, and reading both handwritten and printed text. These findings underscore the potential of our VISA system as an essential aid for the visually impaired. Comparing with existing systems and solutions, our VISA system stands out in terms of all-round effectiveness, versatility, ease of interaction, and vision-related tasks such as object recognition and reading texts.

Throughout this paper, we have demonstrated the effectiveness of the VISA system in indoor environments for everyday activities. However, this system can be expanded and integrated with further advancements in AI. One potential expansion for the VISA system is to provide contextual information about the surrounding environment. While this task is challenging for object recognition algorithms, ongoing advancements in AI technology will enable the VISA system to deliver increasingly refined and intuitive assistance to visually impaired users. For instance, the integration of Large Language Models (LLMs) for picture-to-text translation could allow users to access richer and more detailed information. Additionally, improvements in algorithms and software are possible for the VISA system. Notable examples include a more optimized source code adapted to the Jetson Orin Nano architecture, and an improved depth estimation algorithm based on histogram clustering. Lastly, while the current VISA system may be limited in assisting with outdoor activities, integrating a GPS- and roadmap-based outdoor navigation subsystem could further expand the range of tasks that the VISA system can handle.

## Figures and Tables

**Table 1:** Comparison of networked navigation systems for the visually impaired.

Name	RFID	NFC	BLE	UWB	Infrared
**Principle of Operation**	Tag–Reader	Tag–Reader	Beacon–Receiver (nLoS)	Beacon–Receiver (nLoS)	Beacon–Receiver (LoS)
**Typical Range**	<1 m (passive) >40 m (active) [22,23]	<20 cm (theoretical) <10 cm (actual) [16]	Up to 75 m [26]	Up to 90 m [25]	About 20 m [27]
**Accuracy**	Moderate [16]	Moderate	High	High	Low [28]
**Cost**	Low to Moderate	Low to Moderate	Moderate to High	High	Moderate
**Notes**	Active tags have longer range [23], but needs power supply	Very poor range NFC-capable smartphones can be used [24]	Needs power supply; lacks direction information	Needs power supply; lacks direction information	Needs power supply

**Table 3:** Performance comparison of YOLOv8 variants running on Jetson Orin Nano.

Model	Neural Network Model Parameters (M)	Average Frame Time (ms)	Average FPS	Board Power Consumption (W)	Wattmeter Power Consumption (W)
YOLOv8n (Nano)	3.2	24.12	41.46	6.5	9.9
YOLOv8s (Small)	11.2	27.45	36.43	7.3	11.1
YOLOv8m (Medium)	25.9	62.63	15.97	8.6	12.8
YOLOv8l (Large)	43.7	102.17	9.79	9.5	14.2
YOLOv8x (eXtreme)	68.2	155.59	6.43	10.1	14.7

**Table 4:** ArUco marker database sample.

Node ID	Node Name	Possible Paths	Coordinates
1	Room A	2	[0, 0]
2	Entrance	1, 3, 4	[8, 0]
3	Room B	2	[16, 0]
4	Hall	2, 5, 6, 8	[8, 8]
5	Elevator	4, 7	[0, 8]
6	Women’s Restroom	4	[16, 8]
7	Room C	5, 8	[0, 16]
8	Room D	4, 7, 9	[8, 16]
9	Men’s Restroom	8	[16, 16]

**Table 5:** Graph structure for indoor positioning.

Node ID	Coordinates	Direction
4	(60, −5)	(0, −1)
8	(45, −90)	(0, 1)
996	(10, −15)	(1, 0)

## Data Availability

The data presented in this study are available on request from the corresponding author.

## Abbreviations

The following abbreviations are used in this manuscript:

VISA	Visually impaired spatial awareness
AR	Augmented reality
HMI	Human–machine interface
TTS	Text-to-speech
STT	Speech-to-text
SoC	System-on-chip
GPS	Global positioning system
LoS	Line of sight
nLoS	Non-line of sight
RFID	Radio frequency identification
NFC	Near-field communication
UWB	Ultra-wideband
BLE	Bluetooth low energy
RGB-D	Red–green–blue-depth
QR	Quick response
ArUco	Augmented Reality University of Cordoba
COCO	Common objects in context
FPS	Frames per second
LLM	Large language model
